# Malaria Coinfections Worldwide: An Umbrella Systematic Review of Prevalence and Epidemiological Patterns

**DOI:** 10.3390/tropicalmed11070206

**Published:** 2026-07-22

**Authors:** Víctor Juan Vera-Ponce, Jhosmer Ballena-Caicedo, Holly E. Delgado-Toro, Adriana Mishell Yoplac-Oyarce, Lily Mabel Portal-Valqui, Fiorella E. Zuzunaga-Montoya

**Affiliations:** Facultad de Medicina (FAMED), Universidad Nacional Toribio Rodríguez de Mendoza de Amazonas (UNTRM), Amazonas 01001, Peru; 7330178022@untrm.edu.pe (J.B.-C.); 7694394622@untrm.edu.pe (H.E.D.-T.); 7342299531@untrm.edu.pe (A.M.Y.-O.); 7285300522@untrm.edu.pe (L.M.P.-V.); fiorella.zuzunaga@untrm.edu.pe (F.E.Z.-M.)

**Keywords:** malaria, coinfection, Plasmodium, systematic review, communicable diseases, epidemiology, helminth infections, HIV infections, dengue, viral diseases, bacterial infections

## Abstract

Malaria coinfections with other infectious agents represent a clinically relevant epidemiological challenge, but evidence remains fragmented across individual systematic reviews. This umbrella systematic review synthesized review-level evidence on the prevalence and epidemiological patterns of concurrent malaria coinfections in human populations. PubMed/MEDLINE, Scopus, Web of Science, Embase, and LILACS/BVS were searched for systematic reviews published from 1 January 2000 to March 2026. Risk of bias, certainty of evidence, and primary-study overlap were assessed using ROBIS, an adapted GRADE framework for prevalence evidence, and citation-matrix/corrected-covered-area methods, respectively. Twenty-one systematic reviews were included, of which 16 contributed meta-analytical prevalence estimates. Among acute-pattern coinfections, review-level prevalence estimates ranged from 3.0% (95% CI: 2.0–5.0) for malaria–influenza to 21.7% (95% CI: 18.7–25.1) for malaria–Ebola virus disease. Among chronic or persistent-pattern coinfections, estimates ranged from 6.0% (95% CI: 4.0–7.0) for malaria–hepatitis B to 50.0% (95% CI: 28.0–72.0) for malaria–human African trypanosomiasis; the latter reflects Plasmodium positivity among patients with human African trypanosomiasis and should not be interpreted as a population-level prevalence. Sub-Saharan Africa was the most represented region. Certainty of evidence was generally low or very low, largely because of substantial heterogeneity, diagnostic variability, geographic concentration, and overlap among some source reviews. Malaria coinfections are clinically and epidemiologically relevant in endemic settings, but available prevalence estimates should be interpreted as context-dependent review-level summaries rather than globally comparable burden estimates.

## 1. Introduction

Malaria continues to be one of the most relevant infectious diseases worldwide, with an estimated 282 million cases and 610,000 deaths in 2024 according to the World Malaria Report 2025 [1]. This disease, caused by protozoa of the genus Plasmodium, remains a persistent threat to global public health, particularly in settings with poverty, limited health infrastructure, and climatic conditions conducive to vector transmission. In parallel, infectious diseases such as dengue, schistosomiasis, HIV/AIDS, and leptospirosis share geographic spaces and risk factors with malaria, favoring concurrent infections. This clinical phenomenon complicates differential diagnosis, alters symptomatic presentation, and may modify clinical outcomes [2]. A comprehensive review of malaria coinfections is therefore necessary to understand their epidemiological distribution and to support locally adapted diagnostic reasoning and surveillance.

Over the past two decades, a rise in reports of malaria coinfections with various infectious agents has been observed, attributed to multiple interrelated factors. Among these, the geographic expansion of viral diseases such as dengue, the growth of overlapping endemic zones, population mobility, climate change, and limitations in diagnostic surveillance systems stand out [3,4]. Evidence has shown that certain coinfections, such as malaria and dengue, are increasing in prevalence in regions like Central Africa. In contrast, others, such as coinfection with intestinal helminthiasis or schistosomiasis, are particularly common in rural communities with limited access to sanitation [5]. These scenarios generate significant clinical and health consequences, ranging from misdiagnoses to the administration of inadequate treatments, with a negative impact on already overburdened health systems [6]. This epidemiological evolution demands an updated synthesis of evidence to better understand the magnitude and distribution of these coinfections.

Despite the growing number of primary studies and individual systematic reviews, the literature presents important gaps and methodological heterogeneity. Many reviews are limited to a specific coinfection or particular region, making cross-sectional comparison of results difficult. Additionally, there are substantial discrepancies in reported coinfection rates, which are derived from variations in diagnostic methods, inclusion criteria, and the studied populations [7]. For example, while some studies indicate a positive association between malaria and certain parasitic coinfections, others suggest antagonistic interactions or protective effects, generating controversy and confusion in clinical interpretation [8]. Likewise, few studies have comprehensively addressed the topic, comparing multiple types of coinfection simultaneously. These limitations underscore the need for a systematic review of reviews that consolidates evidence rigorously and allows identification of consistent patterns at the global level.

Previous systematic reviews have generally focused on individual malaria coinfections, specific pathogen groups, or restricted geographic settings. This has limited cross-pathogen comparison and has made it difficult to identify broader epidemiological patterns, methodological limitations, and evidence gaps across the field. An umbrella review is therefore appropriate because it synthesizes evidence at the level of systematic reviews, allowing comparison of review-level prevalence estimates, risk of bias, heterogeneity, diagnostic approaches, certainty of evidence, primary-study overlap, and geographic concentration across multiple malaria coinfection syndromes. Rather than proposing universal screening for all possible pathogens, this review aims to inform locally adapted diagnostic reasoning in endemic settings, where additional testing should be guided by local pathogen circulation, seasonality, clinical severity, host risk factors, and atypical evolution after malaria diagnosis.

Therefore, the objective of this umbrella review (UR) is to synthesize available review-level evidence on the prevalence of concurrent coinfections between malaria and other infectious diseases (viral, bacterial, and parasitic) in human populations, based on published systematic reviews (SRs) and meta-analyses. The aim is to identify infectious agents most frequently reported with malaria, summarize their review-level prevalence estimates, and describe variation according to region, Plasmodium species, affected population, diagnostic methods, and certainty of evidence. Secondary aims were to summarize reported clinical outcomes, assess methodological quality using ROBIS, evaluate primary-study overlap among comparable reviews, and identify research gaps relevant to epidemiological surveillance and clinical practice.

## 2. Materials and Methods

### 2.1. Study Design

**Figure 1:**
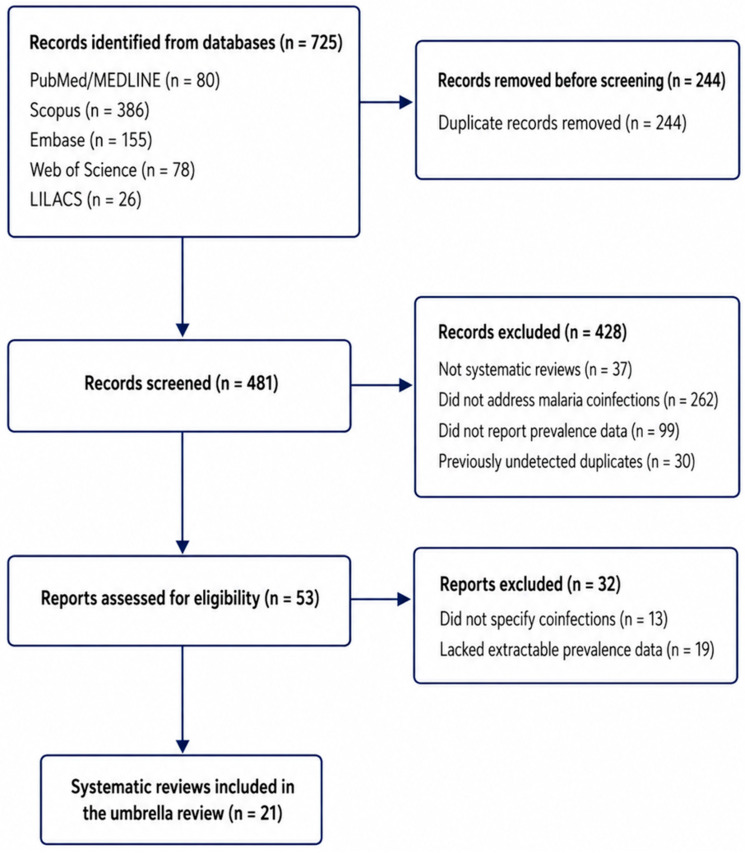
PRISMA 2020 flow diagram of study selection.

An umbrella systematic review of systematic reviews (SRs), with or without meta-analysis, was conducted to evaluate the prevalence of concurrent malaria coinfections with other infectious diseases in human populations. The review was conducted and reported in accordance with the PRISMA 2020 statement [9], adapted to the umbrella review design and guided by methodological recommendations for overviews of reviews [10]. A completed PRISMA 2020 checklist is provided as Supplementary Material S2, and the study-selection process is presented in the PRISMA 2020 flow diagram (Figure 1). The review protocol was not prospectively registered in PROSPERO or another public registry. No public protocol was available; therefore, protocol non-registration was treated as a transparency limitation and is explicitly addressed in the Limitations section. The synthesis plan was descriptive and review-level; no de novo pooled meta-analysis was planned or performed.

### 2.2. Search Strategy

A comprehensive and systematic search strategy was designed to retrieve relevant systematic reviews published from 1 January 2000 to March 2026, without language restrictions. The databases consulted were PubMed/MEDLINE, Scopus, Web of Science (including SciELO), Embase, and LILACS/BVS, in accordance with the Cochrane Handbook recommendations for systematic reviews. The LILACS/BVS component was updated on 1 June 2026 in the BVS Regional Portal using terms harmonized with the other databases for systematic reviews, meta-analyses, umbrella reviews, and overviews of reviews.

For the development of the search strategy, combinations of controlled and free-text terms were used, primarily related to “malaria”, “Plasmodium”, “coinfection”, “systematic review”, “meta-analysis”, and “umbrella review”. Boolean operators (“AND”, “OR”) were applied to structure the strategies. The complete search strategies for each database, including the updated LILACS/BVS strategy, are presented in Supplementary Material S1.

### 2.3. Selection Criteria

Only systematic reviews reporting prevalence estimates of concurrent malaria-infection co-occurrence were included. For acute infections, concurrency was defined as laboratory- or clinically confirmed malaria and a second infectious disease diagnosed during the same febrile episode or clinical encounter. For chronic or persistent infections, including HIV/AIDS, hepatitis B virus infection, schistosomiasis, and soil-transmitted helminthiasis, concurrency was defined as confirmed malaria occurring in individuals with documented evidence of ongoing or established infection by the second pathogen at or near the time of malaria assessment. Therefore, the term “concurrent coinfection” was used operationally and does not imply simultaneous acquisition, identical incubation periods, latency status, or causal interaction between pathogens. Reviews with or without meta-analysis were accepted, provided they offered prevalence estimates or allowed clear extraction from the reported data. No restrictions were imposed by age, sex, Plasmodium species, or geographic region.

Studies that were not systematic reviews (such as narrative reviews, bibliographic reviews, scoping reviews, or bibliometric reviews) were excluded, as well as letters to the editor, case reports, clinical series, and reviews conducted exclusively in animal models. Reviews that addressed malaria coinfections without specifying whether they met the operational definition of concurrent malaria-infection co-occurrence, or that lacked prevalence data, were also excluded. Likewise, studies whose full text was not available were excluded.

### 2.4. Temporality of Coinfections

For interpretative purposes, coinfections were grouped according to the predominant natural history of the non-malarial infection rather than as a strict biological dichotomy. Acute-pattern coinfections included infections usually presenting as temporally circumscribed febrile illnesses, such as dengue, influenza, COVID-19, Ebola virus disease, leptospirosis, bacteremia, salmonellosis, and severe pneumonia. Chronic or persistent-pattern coinfections included infections characterized by prolonged carriage, persistence, or repeated exposure, such as HIV/AIDS, hepatitis B virus infection, schistosomiasis, soil-transmitted helminthiasis, and human African trypanosomiasis. This grouping was used only to structure the synthesis and interpretation of prevalence estimates. It should not be interpreted as evidence that infections were acquired simultaneously, that temporality was identical across pathogens, or that causal interaction was demonstrated.

### 2.5. Study-Selection Process

Search results were exported to a reference manager and subsequently uploaded to the Rayyan QCRI platform to facilitate identification and removal of duplicates. Two reviewers independently evaluated titles and abstracts to determine the initial eligibility of articles, and then reviewed the full text of selected ones. The process was conducted anonymously and in parallel, resolving discrepancies through discussion and, when necessary, with the intervention of a third reviewer. The PRISMA diagram was followed to document the selection flow.

During the selection process, it was verified that each SR specified diagnostic confirmation of malaria and the coinfecting disease according to the operational definition of concurrent coinfection described above. Reviews were included if they presented clearly defined clinical or laboratory criteria and reported prevalence or pooled estimates from primary studies.

### 2.6. Data Extraction

Data extraction was performed by two reviewers using a standardized matrix in Microsoft Excel 2023. From each included review, the following were extracted: author and year, coinfecting disease, number of included primary studies, total sample size, geographic region, evaluated population, type of Plasmodium, diagnostic method for malaria and coinfection, operational definition of coinfection, and, primarily, point prevalence of coinfection with its 95% confidence interval (95% CI). Relevant methodological comments and notes on study quality were also recorded.

Data were cross-verified between reviewers, and any discrepancy was resolved by returning to the original text. Information was organized by type of coinfecting agent (viruses, bacteria, and parasites) and by geographic region, to present a clear and structured synthesis of the findings.

### 2.7. Risk-of-Bias Assessment

The methodological quality of included systematic reviews was evaluated using the ROBIS (Risk of Bias in Systematic Reviews) tool, specifically designed to assess risk of bias in systematic reviews used as a basis for secondary syntheses, such as URs [11]. This tool consists of three phases: evaluating key domains, including the formulation of eligibility criteria, the search and study-selection process, data collection and evaluation, and the synthesis of findings.

Two reviewers independently and in parallel applied the ROBIS tool. Any discrepancy was resolved through consensus. Reviews were classified as low or high risk of bias, particularly in consideration of the methodological quality of the final synthesis. The differences observed between reviews with low and high risk of bias were primarily due to limitations in the quantitative or narrative analysis of findings, as well as the lack of assessment of heterogeneity or certainty in the results.

### 2.8. Narrative Synthesis, Overlap Assessment, and Certainty of Evidence

For each included review, information corresponding to the type of coinfection, geographic region, number of primary studies and patients, point prevalence of coinfection with its 95% confidence interval, heterogeneity, and risk of bias was extracted and organized. Estimates reported in the source reviews were retained as review-level estimates; no de novo pooled meta-analysis was conducted in this umbrella review. Information was grouped by type of coinfecting pathogen and by predominant infection pattern, allowing identification of frequency, heterogeneity, diagnostic, and geographic distribution patterns.

Results were organized in tabular summaries and comparative figures when appropriate. Because several source reviews synthesized highly heterogeneous primary studies, pooled prevalence estimates were treated as descriptive review-level summaries rather than as directly comparable global estimates. This narrative presentation was designed to avoid introducing additional artifacts from heterogeneity among the included reviews.

Primary-study overlap among systematic reviews was assessed using the corrected covered area (CCA) method [12]. CCA was calculated only for groups of reviews addressing comparable pathogen-specific objectives and with identifiable primary-study lists. The formula used was CCA = (N − r)/[(r × c) − r], where N is the total number of primary-study occurrences across reviews, r is the number of unique primary studies, and c is the number of reviews in the matrix. CCA was interpreted as slight (0–5%), moderate (>5–10%), high (>10–15%), or very high (>15%) overlap. CCA was not calculated for pathogen groups represented by a single review, for review pairs with non-comparable objectives, or when complete primary-study lists were unavailable.

Certainty of evidence for each review-level malaria coinfection prevalence estimate was appraised using an adapted GRADE framework for prevalence evidence. The assessment considered six domains: ROBIS overall risk of bias, heterogeneity, diagnostic directness for malaria and the coinfecting pathogen, precision of the pooled estimate or reported range, geographic representativeness, and publication-bias or small-study concerns. Overall certainty was rated as high, moderate, low, or very low. Reviews without pooled prevalence estimates or with only narrative/range-based synthesis were not upgraded beyond low certainty.

## 3. Results

### 3.1. Article Selection Process

The initial database search yielded 725 records from PubMed/MEDLINE (n = 80), Scopus (n = 386), Embase (n = 155), Web of Science (n = 78), and LILACS/BVS (n = 26). No additional records were identified through other sources. After removing 244 duplicates, 481 records were screened by title and abstract. Of these, 428 were excluded because they were not systematic reviews (n = 37), did not address coinfections with malaria (n = 262), did not report prevalence data (n = 99), or were duplicate records detected during screening (n = 30). The remaining 53 reports were sought for retrieval and all were obtained; these 53 full-text reports were assessed for eligibility. Thirty-two full-text reports were excluded because they did not specify coinfections (n = 13) or lacked extractable prevalence data (n = 19). Ultimately, 21 systematic reviews met all inclusion criteria and were included in this umbrella review, of which 16 contributed meta-analytical prevalence estimates and 5 contributed narrative or descriptive evidence only [3,4,5,6,13,14,15,16,17,18,19,20,21,22,23,24,25,26,27,28,29].

A targeted post hoc rerun of the LILACS/BVS component was performed on 1 June 2026 in the BVS Regional Portal using harmonized systematic-review and meta-analysis terms. When restricted to the LILACS Plus collection, the search retrieved six records, corresponding to five unique records after one duplicate. No additional eligible systematic review was identified; consequently, the final number of included reviews remained unchanged.

### 3.2. General Characteristics of Included Studies

Twenty-one systematic reviews published between 2014 and 2025 were included in this analysis (See Table 1). Of these, 16 conducted meta-analyses (including one that additionally incorporated meta-regression [3], while 5 studies did not perform meta-analysis, limiting themselves to narrative syntheses or descriptive analyses [4,13,14,23,28]. The number of primary studies included per review ranged from 6 to 104, with sample sizes ranging from 1048 to over 73,000 individuals, indicating notable methodological and population heterogeneity.

The most frequently evaluated coinfections were those of malaria with intestinal helminthiasis [5,19,27,29], followed by viral coinfections such as dengue [3,14], COVID-19 [4,20], HIV [15,23], influenza virus [26], and Ebola virus disease [21]. Additionally, studies on coinfection with enteric bacteria [13,18,25], leptospirosis [17], hepatitis B [16], African trypanosomiasis [24], and respiratory diseases such as severe pneumonia [6] were included. The review by Cerilo-Filho et al. (2024) [28] furthermore addressed multiple coinfections with dengue, chikungunya, Zika, and yellow fever, reporting prevalence ranges by region.

The most represented geographic regions were sub-Saharan Africa, Southeast Asia, and South America, with populations that included children, febrile outpatients, immunocompromised adults, and hospitalized individuals. In all studies, confirmatory methods for malaria were applied through microscopy, rapid diagnostic tests (RDTs), or PCR. Coinfecting diseases were diagnosed with methods appropriate to each pathogen, including ELISA, culture, molecular tests, rapid serological tests, and, in some cases, clinical and imaging criteria (such as those established by Integrated Management of Childhood Illness or IMCI for pneumonia).

### 3.3. Assessment of Heterogeneity in Meta-Analyses

Regarding heterogeneity, I^2^ statistics were extracted from each study when available, particularly from meta-analyses. The analysis revealed that I^2^ values ranged from 36% (Edwards et al. 2021, Ebola coinfection) [21] to 99.97% (Abebe et al. 2025, schistosomiasis [*S. mansoni*] coinfection) [29], with 17 out of 21 studies reporting I^2^ values above 75%. The highest heterogeneity levels were observed in studies evaluating chronic coinfections, particularly those involving helminthiasis and AIDS (I^2^ > 95% in most cases). Studies of coinfections showed variable heterogeneity levels, ranging from moderate (I^2^ = 77.5% for influenza coinfection) to very high (I^2^ = 99.42% for non-typhoidal salmonellosis coinfection). This substantial heterogeneity reflects methodological diversity across primary studies, including differences in diagnostic approaches, population characteristics, geographic contexts, and local epidemiological factors influencing coinfection patterns. Given this magnitude of inconsistency, pooled prevalence estimates were interpreted as broad review-level summaries rather than precise or directly comparable measures of coinfection burden. High I^2^ values likely reflect both genuine epidemiological variability and methodological diversity across primary studies, including differences in diagnostic platforms, study settings, population risk profiles, malaria endemicity, coinfecting-pathogen endemicity, and clinical case definitions. Therefore, the estimates presented below should not be interpreted as a ranking of pathogens or as globally transferrable prevalence values. Their primary value lies in identifying where evidence is concentrated, which coinfections have repeatedly been studied, and where interpretation is constrained by inconsistency.

The five studies that did not perform meta-analysis [4,13,14,23,28] provide complementary evidence through reporting individual prevalences, analysis by clinical subgroups, clinical manifestations, severe outcomes, and geographic distribution of coinfections. Although they do not contribute pooled estimates, their inclusion enriches qualitative understanding of epidemiological and clinical patterns of malaria coinfection.

### 3.4. Risk-of-Bias Analysis

The risk of bias of the 21 systematic reviews included in this umbrella review was evaluated using the ROBIS tool (see Table 2). Most reviews were rated as having a low risk of bias in all domains related to eligibility criteria, study identification and selection, and data collection and evaluation.

However, seven reviews [3,6,13,24,25,26,27] were considered to have high risk of bias, primarily due to limitations in the synthesis and findings domain. Notably, six of these reviews conducted meta-analyses but had methodological limitations in their quantitative synthesis or inadequate assessment of heterogeneity. Only one high-risk review did not perform meta-analysis, relying exclusively on narrative synthesis despite having quantitative data available. Although their study-selection and evaluation processes were generally rigorous, the limited analytical depth and incomplete handling of heterogeneity reduced the reliability of their findings.

### 3.5. Primary-Study Overlap and Certainty of Evidence

CCA could be calculated for four comparable clusters. The malaria–dengue matrix, using DENV-specific rows from the broader arbovirus reviews, showed very high overlap (Gebremariam 2023, Cerilo-Filho 2024, and Salam 2018: N = 128, r = 80, c = 3, CCA = 30.0%). The malaria–Schistosoma mansoni matrix showed high overlap (Abebe 2025 and Setegn 2024: N = 24, r = 21, CCA = 14.3%). The malaria–COVID-19 matrix showed slight overlap (Wilairatana 2021 and Mohamed 2024: N = 31, r = 30, CCA = 3.3%). A broader sensitivity matrix for malaria–helminth/STH reviews showed moderate overlap (Afolabi 2021, Degarege 2016, and Boltena 2021: N = 76, r = 64, CCA = 9.4%). These findings were considered when interpreting evidence so that highly overlapping reviews did not receive disproportionate weight. Full details are provided in Supplementary Material S3.

The certainty assessment is summarized in Table 3. Certainty was generally low or very low, primarily because most pooled estimates showed substantial heterogeneity, diagnostic algorithms differed across primary studies, several estimates were geographically concentrated, and narrative reviews did not provide pooled precision. Moderate certainty was assigned only when the review had low ROBIS risk, direct diagnostic confirmation, comparatively lower heterogeneity, and precise confidence intervals.

### 3.6. Quantitative Synthesis of Malaria Coinfection Prevalence

**Figure 2:**
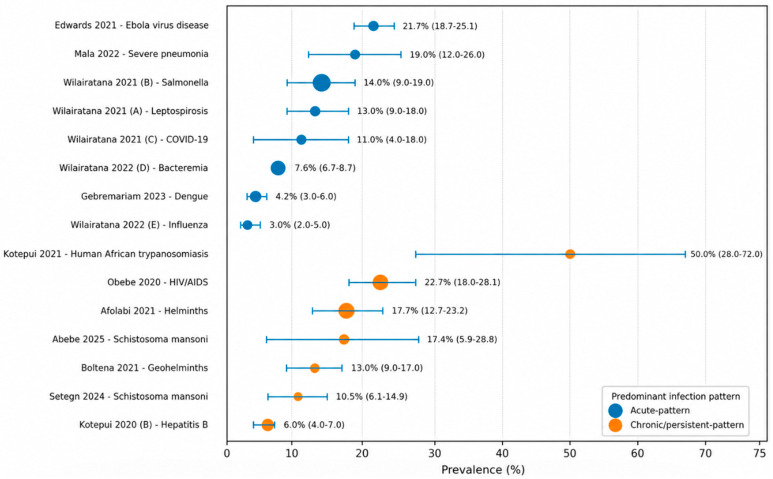
Review-level pooled prevalence estimates of concurrent malaria coinfections according to predominant infection pattern. Estimates were extracted from the included systematic reviews and meta-analyses and were not recalculated de novo. Because most underlying meta-analyses reported substantial heterogeneity and several estimates had wide confidence intervals, the figure should be interpreted as a descriptive evidence map rather than as a direct ranking of coinfection prevalence across pathogens. Point size represents the number of primary studies included in each source review.

Because no de novo meta-analysis was conducted in this umbrella review, the prevalence estimates displayed in Figure 2 correspond to estimates extracted from the included systematic reviews and meta-analyses. They are presented for descriptive comparison only and should be interpreted in light of the heterogeneity, diagnostic variability, denominators, and population differences reported by each source review. Prevalences ranged from 3.0% (95% CI: 2.0–5.0%) for malaria–influenza [26] to 50.0% (95% CI: 28.0–72.0%) for malaria–human African trypanosomiasis among patients with human African trypanosomiasis [24]. High prevalences were also identified in coinfection with severe pneumonia (19.0%, 95% CI: 12.0–26.0%) [6], non-typhoidal salmonellosis (14.0%, 95% CI: 9.0–19.0%) [18], leptospirosis (13.0%, 95% CI: 9.0–18.0%) [17], and COVID-19 (11.0%, 95% CI: 4.0–18.0%) [20]. Coinfection with bacteremia showed a moderate prevalence of 7.6% (95% CI: 6.7–8.7%) [25], while malaria–dengue presented a prevalence of 4.2% (95% CI: 3.0–6.0%) [3].

Notable findings included Ebola virus disease coinfection at 21.7% (95% CI: 18.7–25.1%) [21], AIDS (22.7%, 95% CI: 18.0–28.1%) [15], and intestinal helminthiasis (17.7%, 95% CI: 12.7–23.2%) [19]. Coinfections with schistosomiasis (*S. mansoni*) varied between 10.5% (95% CI: 6.1–14.9%) [27] and 17.4% (95% CI: 5.9–28.8%) [29], while with soil-transmitted helminthiasis they reached 13.0% (95% CI: 9.0–17.0%) [22]. The lowest prevalence was observed with hepatitis B (6.0%, 95% CI: 4.0–7.0%) [16]. These estimates evidenced important geographic differences, with higher proportions reported in sub-Saharan Africa and endemic regions of Southeast Asia. The estimate for malaria–human African trypanosomiasis should be interpreted with particular caution because it was derived from patients with confirmed or suspected HAT rather than from a general febrile or community population. Therefore, it reflects Plasmodium positivity among HAT patients and should not be directly compared with estimates derived from broader malaria-endemic populations.

The size of the point in the graph represents the number of studies included in each review, allowing for the simultaneous visualization of the robustness and weight of evidence. The most studied coinfections were malaria–Salmonella (81 studies) and malaria–helminths (55 studies). The graph highlights how different studies with similar prevalences evaluate different pathogens (Salmonella vs. Leptospira) [17,18], which emphasizes the importance of contextualizing each prevalence according to etiology and the studied population.

**Figure 3:**
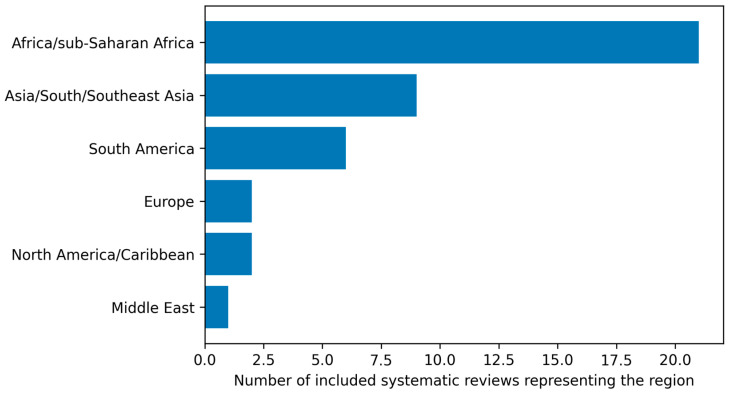
Review-level geographic coverage of included systematic reviews. Counts indicate source-review representation by region and are not de-duplicated counts of primary studies or participants.

Figure 3 presents review-level geographic coverage of the malaria coinfection evidence base. Because several primary studies may appear in more than one systematic review, these counts reflect the number of included reviews representing each region and should not be interpreted as counts of unique primary studies or unique participants.

## 4. Discussion

### 4.1. Main Findings and Their Epidemiological Context

This UR reveals a complex epidemiological landscape of malaria-associated coinfections, with distinctive patterns according to pathogen type, denominator, geographic distribution, and diagnostic approach. Among acute-pattern coinfections, review-level prevalence estimates ranged from 3.0% (95% CI: 2.0–5.0%) for malaria–influenza to 21.7% (95% CI: 18.7–25.1%) for malaria–Ebola virus disease. Among chronic or persistent-pattern coinfections, estimates ranged from 6.0% for malaria–hepatitis B to 50.0% for malaria–human African trypanosomiasis. The trypanosomiasis estimate must be interpreted as Plasmodium positivity among patients with HAT, not as a population-level prevalence estimate. Overall, these findings suggest that malaria coinfections are epidemiologically relevant in endemic settings, but their magnitude is highly context-dependent.

The differentiation between acute-pattern and chronic or persistent-pattern coinfections was used as an interpretative framework rather than as a strict biological taxonomy. Acute-pattern coinfections, such as malaria–dengue, malaria–pneumonia, malaria–bacteremia, malaria–leptospirosis, or malaria–influenza, are more likely to be shaped by outbreaks, seasonality, febrile illness surveillance, and short diagnostic windows. Chronic or persistent-pattern coinfections, such as malaria–HIV, malaria–hepatitis B, and malaria–helminth infections, more often reflect prolonged pathogen carriage, repeated exposure, and shared socio-environmental determinants. This distinction is useful for organizing evidence but does not establish simultaneous acquisition, uniform temporality, or causal interaction.

The geographic distribution of coinfections shows differentiated patterns but with predominant concentration in sub-Saharan Africa, where high malaria endemicity, elevated HIV prevalence, limited access to sanitation, and limited health infrastructure converge [30]. This region presents the highest prevalence of malaria–HIV coinfection (22.7%) [15], and malaria–helminths (17.7%) [19], configuring a scenario of infectious polycausality that complicates healthcare delivery. Southeast Asia emerges as the second region with the highest frequency of coinfections, particularly notable for the presence of malaria–dengue and malaria–leptospirosis [2,30]. In contrast, Latin America presents lower general prevalences, although with important foci of malaria–dengue coinfection in the Amazon basin [31].

However, several geographic limitations should be acknowledged in our findings. The overrepresentation of studies from sub-Saharan Africa reflects both the region’s disproportionate malaria burden (approximately 95% of global cases) and the concentration of research efforts in endemic areas. Conversely, regions such as Oceania are underrepresented because malaria transmission is geographically limited to specific countries like Papua New Guinea and certain Pacific islands. Similarly, temperate regions where malaria is not endemic naturally lack relevant coinfection studies. Therefore, caution should be exercised when generalizing our findings to underrepresented geographic areas, and the prevalence estimates presented should be interpreted within their specific epidemiological contexts rather than as universal values applicable to all regions.

Seasonality and endemicity probably modify observed coinfection patterns, particularly for arboviruses, leptospirosis, influenza, and malaria itself. However, these variables were inconsistently reported across the included reviews, limiting formal comparison. Similarly, severity outcomes were not uniformly available; where reported, severe pneumonia, bacteremia, Ebola virus disease, and HIV-associated coinfections showed clinically relevant outcome signals, whereas several prevalence-focused reviews provided little or no comparable severity information. Future primary studies and reviews should report season, transmission intensity, clinical setting, and severity using standardized definitions.

The consistent methodological practices observed in most included reviews reflect adequate development of protocols, comprehensive search strategies, and clear presentation of inclusion criteria and evaluation methods. However, the marked variability in reported prevalences not only reflects real epidemiological differences but also significant methodological variations between studies. For example, malaria–bacteremia coinfection oscillated between 6.4% and 7.6% in pediatric hospital studies [13,25], while malaria–COVID-19 prevalence varied between 4% and 18% according to context [20]. These differences can be attributed to factors such as diagnostic criteria employed (microscopy vs. PCR), methodological designs (hospital vs. community studies), and target populations (febrile patients vs. severe cases), as well as local ecological factors that modulate simultaneous transmission [32,33]. Of particular relevance is the observation that studies with more sensitive diagnostic methodologies (PCR) tend to report prevalences up to 2–3 times higher than those based exclusively on microscopy or rapid tests [34], raising questions about the underdiagnosis of coinfections in resource-limited settings.

### 4.2. Implications for Clinical Diagnosis

The concurrent presence of malaria and another infection can alter clinical presentation, but the degree and direction of this effect vary across pathogens, populations, and diagnostic settings. Malaria–dengue coinfection, for example, may be associated with overlapping febrile illness, thrombocytopenia, hepatic enzyme elevation, or hemorrhagic manifestations in some settings [14,35]. These patterns can lead to incomplete or erroneous diagnoses when one positive test result is treated as a sufficient explanation for the entire clinical syndrome.

The analyzed studies reveal that between 14% and 36% of malaria cases present coinfections that could go unnoticed under monocausal diagnostic paradigms [15,20]. This phenomenon is especially problematic in endemic areas where “malaria presumption” may dominate clinical reasoning and lead to attribution of all symptomatology to a single agent [15,20]. D’Acremont et al. [36] demonstrated that up to 30% of patients with confirmed malaria diagnosis also presented another infectious etiology that explained part of the symptomatology, a finding confirmed by meta-analyses included in our review.

The diagnostic challenge is magnified by symptomatic overlap between pathogens, particularly notable in coinfections such as malaria–leptospirosis, malaria–typhoid fever, or malaria–COVID-19, where fever, headache, myalgia, and gastrointestinal symptoms are common to both entities [37,38]. This situation can lead to scenarios where the identification of one pathogen stops the search for potentially treatable coinfections. The introduction of rapid diagnostic tests for malaria has improved diagnostic accuracy, but paradoxically may have increased this phenomenon by providing a sufficient explanation for the febrile illness [39].

These findings support consideration of locally adapted diagnostic approaches rather than universal screening for all possible pathogens. Additional testing should be guided by local epidemiology, seasonality, clinical severity, atypical laboratory findings, persistence of fever despite appropriate antimalarial therapy, and host risk factors such as pregnancy or immunosuppression. In resource-limited settings, this risk-based approach is more defensible than indiscriminate multiplex testing because it links diagnostic expansion to epidemiological plausibility and clinical need.

### 4.3. Impact of Coinfections on Clinical Outcomes

Malaria coinfections may modify clinical evolution, but severity signals should be interpreted cautiously because the included reviews differed substantially in population, setting, diagnostic criteria, and outcome definitions. Some reviews reported clinically important associations with severe outcomes, particularly malaria–severe pneumonia, malaria–bacteremia, Ebola virus disease with malaria parasitemia, and malaria–HIV coinfection [6,13,15,21,25]. However, this umbrella review synthesized prevalence-focused evidence and cannot determine whether coinfection independently caused worse outcomes after accounting for confounding by age, comorbidity, transmission intensity, or health-system access.

Biological interactions between pathogens may be synergistic or antagonistic, but the available review-level evidence is more consistent for clinical co-occurrence than for mechanism-specific causality. In malaria–HIV coinfection, impaired cellular immunity may favor higher parasitemia and poorer clinical control, while acute malaria episodes may transiently increase immune activation and HIV replication [15,40]. In helminthic coinfections, immunomodulation may alter parasitemia or anemia profiles, but findings are not uniformly protective or harmful and likely vary by helminth species, intensity, chronicity, and host background [5,19,41].

Specific populations may be particularly vulnerable to clinically relevant coinfections, including young children, pregnant women, hospitalized patients, and immunocompromised individuals. Nevertheless, the evidence does not justify a single universal diagnostic or therapeutic protocol for all coinfections. Instead, diagnostic suspicion and management should be adapted to patient risk profile, local epidemiology, severity, and the availability of reliable confirmatory tests.

### 4.4. Pathophysiological Mechanisms of Interactions Between Malaria and Coinfections

The biological interpretation of malaria coinfections should be considered hypothesis-generating because the present umbrella review synthesized prevalence estimates and did not directly evaluate mechanistic causality. Nevertheless, several plausible interaction pathways may help contextualize the clinical patterns reported in the included reviews. In malaria–HIV coinfection, impaired cellular immunity may favor higher parasitemia and poorer clinical control, while acute malaria episodes may transiently increase immune activation and HIV replication. In malaria–bacterial coinfections, particularly invasive bacteremia and non-typhoidal Salmonella, malaria-associated hemolysis, endothelial activation, and transient immune dysfunction may increase susceptibility to invasive bacterial disease.

For helminthic coinfections, the direction of interaction is less consistent. Chronic helminth infections may modulate inflammatory responses and have been associated in some studies with altered parasitemia or anemia profiles; however, these findings should not be interpreted as uniformly protective or harmful. Arboviral and other acute febrile coinfections may worsen diagnostic ambiguity because of overlapping symptoms rather than because a specific immune pathway has been consistently demonstrated. Overall, mechanistic hypotheses are useful for framing clinical suspicion and future research, but the prevalence-based evidence summarized here cannot establish pathogen–pathogen causality.

### 4.5. Potentially Underestimated Coinfections and Research Gaps

Tuberculosis–malaria coinfection represents a relevant but insufficiently synthesized area. Both diseases overlap geographically in several endemic regions and may interact through immune activation, anemia, nutritional vulnerability, and treatment-related considerations. However, available systematic evidence remains limited and heterogeneous, preventing robust prevalence comparison with the coinfections included in the quantitative synthesis [42].

Relapsing fever borreliosis is another potentially underrecognized malaria-like coinfection syndrome. It was not included in the quantitative synthesis because no eligible systematic review with extractable malaria–relapsing fever prevalence estimates was identified. However, primary and experimental evidence suggests that relapsing fever Borrelia spp. may be misdiagnosed as malaria in co-endemic settings and may interact biologically with malaria through blood-stage infection dynamics and erythrocyte-related mechanisms [43,44]. This supports its inclusion as a research gap rather than as a prevalence estimate in the present umbrella review.

Emerging arboviruses such as Zika, chikungunya, and yellow fever represent another area with limited systematic evidence, despite their growing relevance in regions such as Latin America and West Africa. Cerilo-Filho et al. [28] mention cases of triple coinfection (malaria–dengue–chikungunya) with particularly severe outcomes, but consistent prevalence estimates and analysis of their distinctive manifestations are lacking.

Invasive fungal coinfections, particularly Histoplasma and Cryptococcus, among immunocompromised patients with malaria remain another potential gap. These pathogens may contribute to severe febrile illness in endemic settings, but they require diagnostic methods that are rarely implemented in malaria coinfection studies; therefore, no prevalence inference can be drawn from the present umbrella review.

Finally, there is a notable gap in research on specific coinfections with *P. vivax*, *P. ovale*, and *P. malariae*, with available evidence concentrated on *P. falciparum*. Considering biological differences between Plasmodium species, particularly *P. vivax*’s tendency to generate hepatic hypnozoites and relapses, it is plausible that interactions with other pathogens present distinctive characteristics that merit specific investigation.

These knowledge gaps evidence the need to broaden the research horizon beyond traditionally studied coinfections, employing advanced diagnostic methodologies and longitudinal approaches that allow characterization of the complete spectrum of relevant coinfections in different epidemiological contexts.

### 4.6. Implications for Public Health and Clinical Practice

The findings of this UR may inform integrated surveillance and clinical awareness in areas where malaria overlaps with other endemic infections. They do not, however, establish the effectiveness or cost-effectiveness of specific diagnostic algorithms, therapeutic prioritization schemes, or ecosystem-based interventions. Surveillance and diagnostic strategies should be adapted to local pathogen circulation, seasonality, clinical setting, available tests, and health-system capacity.

From a public health perspective, the evidence supports moving beyond strictly monocausal interpretations of febrile illness in high-transmission settings. However, implementation of multiplex diagnostic panels, combined prevention packages, or integrated control strategies requires prospective clinical evaluation and context-specific implementation studies before being recommended as standard practice.

### 4.7. Strengths and Limitations

This umbrella systematic review has several strengths, including a broad multisource search strategy without language restrictions, explicit eligibility criteria, independent study selection and data extraction, structured assessment of risk of bias using ROBIS, assessment of primary-study overlap using CCA where appropriate, an adapted certainty-of-evidence assessment, and transparent synthesis of prevalence estimates across predominant infection-pattern categories.

Several limitations must be considered. First, the protocol was not prospectively registered in PROSPERO or another public registry, which limits external verification of some a priori methodological decisions. However, this limitation should be interpreted in the context of the review objective. This umbrella review synthesized prevalence estimates and epidemiological patterns rather than intervention effects, treatment efficacy, or statistically significant associations. Therefore, the main concern related to non-registration was not selective non-publication of “negative” findings, but potential flexibility in eligibility criteria, classification of coinfection patterns, and synthesis decisions. To reduce this risk, we explicitly reported the search strategy, eligibility criteria, study-selection process, data-extraction domains, ROBIS assessment, certainty appraisal, and primary-study overlap assessment. Second, the prevalence estimates were constrained by the methodological quality, diagnostic definitions, and heterogeneity of the included systematic reviews and their primary studies. Differences in malaria diagnostics, diagnostic confirmation of the coinfecting pathogen, clinical setting, population structure, local transmission intensity, and denominator definition likely contributed to the high I^2^ values observed in many included meta-analyses. Consequently, pooled estimates should be interpreted as context-dependent review-level summaries rather than precise or globally transferrable prevalence values.

Third, publication bias and small-study effects could not be consistently assessed at the umbrella-review level because these assessments were incompletely reported across the source reviews. Fourth, the evidence base was geographically imbalanced, with substantial representation of sub-Saharan Africa and fewer data from other endemic regions; therefore, the estimates should not be interpreted as globally generalizable prevalence values. Fifth, although primary-study overlap was addressed using a citation-matrix framework and CCA where possible, exact quantification was not applicable to singleton review clusters, non-comparable review questions, or review groups without complete primary-study lists. Finally, seasonality, endemicity, and severity were inconsistently reported across the included reviews, limiting formal comparison of these effect modifiers.

## 5. Conclusions

This umbrella systematic review summarizes review-level evidence on malaria coinfections across viral, bacterial, and parasitic pathogens. The available evidence suggests that malaria coinfections are clinically and epidemiologically relevant in endemic settings, but prevalence estimates vary widely according to pathogen, population, diagnostic method, geography, denominator, and study design. Therefore, the estimates should be interpreted as context-dependent review-level summaries rather than as globally comparable prevalence values.

The findings support locally adapted diagnostic awareness of coinfections in patients with malaria, particularly when clinical evolution is atypical, severity is disproportionate to parasitemia, fever persists despite appropriate treatment, or local epidemiology suggests co-circulation of other pathogens. However, the evidence synthesized here does not establish universal diagnostic algorithms, therapeutic priorities, or public health interventions. Future studies should improve reporting of temporality, seasonality, diagnostic confirmation, severity, denominators, and primary-study overlap, and should address underrepresented coinfections such as malaria–tuberculosis, malaria–relapsing fever borreliosis, emerging arboviruses, and non-falciparum malaria coinfections.

## Figures and Tables

**Table 1:** Key characteristics of the systematic reviews assessing malaria coinfections.

First Author and Year	Coinfecting Disease	Number of Included Studies	Total Sample Size Analyzed	Target Population	Included Geographic Regions	Plasmodium Type(s) Evaluated	Malaria Diagnostic Method	Coinfecting Disease Diagnostic Method	Heterogeneity Level (I^2^)
Abebe et al., 2025 [29]	Schistosomiasis (*Schistosoma mansoni*)	18	NR	The general population of sub-Saharan Africa	Sub-Saharan Africa	*P. falciparum* (predominant)	Microscopy (mainly), RDT, PCR	Microscopy (mainly)	I^2^ = 99.97%
Setegn et al., 2024 [27]	Schistosomiasis (*Schistosoma mansoni*)	6	2683	General population of Ethiopia	Ethiopia (Amhara and Benishangul Gumuz regions)	*P. falciparum* and *P. vivax*	Microscopy, RDT, PCR	NR	I^2^ = 94.10%
Cerilo-Filho et al., 2024 [28]	Dengue, chikungunya, Zika, and yellow fever	56	52,913	General population	Asia (57.14%), Africa (25%), South America (14.30%), Europe (3.56%)	*P. falciparum*, *P. vivax*, *P. knowlesi*	Thick smear (main), RDT, PCR	ELISA IgM/IgG (main), ELISA NS1, RT-PCR	Not applicable (no meta-analysis)
Mohamed et al., 2024 * [4]	COVID-19	19	2810 (618 cases of coinfection)	Adults and children	Sub-Saharan Africa	*P. falciparum*, *P. vivax*	Microscopy, RDT, PCR	RT-PCR	Not applicable (no meta-analysis)
Gebremariam et al., 2023 [3]	Dengue	22	22,803	Patients with acute undifferentiated fever in Africa	10 African countries	*P. falciparum*	Microscopy, RDT, PCR	ELISA (IgM/NS1), RT-PCR	I^2^ = 95.18%
Mala et al., 2022 [6]	Severe pneumonia	11	20,063 (9738 cases of severe pneumonia and 10,325 cases of malaria)	Mainly children < 5 years	Africa (Mozambique, Tanzania, Kenya, Nigeria, Uganda, Malawi)	Mainly *P. falciparum*	Microscopy, RDT	WHO criteria (IMCI), chest X-ray	I^2^ = 98.97%
Wilairatana et al. (D), 2022 [25]	Bacteremia	51	1583 cases of coinfection	Mainly febrile children	Africa (86.3%) and Asia (11.8%)	Predominantly *P. falciparum*	Microscopy, RDT, or PCR	Blood culture or PCR	I^2^ = 96.64%
Wilairatana et al. (E), 2022 [26]	Influenza	10	22,066 febrile patients (650 coinfected)	Patients with fever	Nigeria, Tanzania, Uganda, Malawi, Ghana, Cambodia, Central African Republic, Kenya	*P. falciparum* (predominant)	Microscopy, RDT, PCR, or combination	RDT, PCR, ELISA, or not specified	I^2^ = 77.5%
Afolabi et al., 2021 [19]	Schistosomiasis (*S. haematobium* and *S. mansoni*)	55 (28 in meta-analysis)	37,559	Children	Sub-Saharan Africa, Southeast Asia, and South America	*P. falciparum*	Microscopy	Urine filtration (*S. haematobium*), Kato Katz (*S. mansoni*)	I^2^ = 98.70%
Wilairatana et al. (A), 2021 [17]	Leptospirosis	15	5838 febrile patients and 421 patients with malaria	Febrile patients and patients with confirmed malaria	India [5], Thailand [3], Cambodia [2], Bangladesh, Jamaica, Malaysia, Tanzania, Venezuela	Not systematically specified for all studies (mentions *P. falciparum* and *P. vivax* in some)	Microscopy (8/15), microscopy/RDT/PCR (3/15), microscopy/RDT, ELISA, MAT, RDT	ELISA (7/15), ELISA/MAT (4/15), PCR (2/15), IFA (1/15), MAT only (1/15), MAT/PCR (1/15)	I^2^ = 83.29%
Wilairatana et al. (B), 2021 [18]	Typhoid fever/non-typhoidal salmonellosis	81	73,775	Febrile patients, pregnant women, and children	Africa and Asia	Not specified, mainly *P. falciparum*	Microscopy, RDT	Widal test, blood culture, stool culture, RDT, molecular methods	I^2^ = 99.42%
Wilairatana et al. (C), 2021 [20]	COVID-19	12	1126 (364 cases of coinfection)	Adults and children	Africa, Asia, and South America	*P. falciparum*, *P. vivax*	Microscopy, RDT, PCR	RT-PCR	I^2^ = 97.07%
Edwards et al., 2021 [21]	Ebola virus disease (EVD)	17 (9 included in meta-analysis)	NR	Confirmed EVD cases	Africa (Guinea, Liberia, Sierra Leone, DRC)	Mainly *P. falciparum* (not specified in all studies)	RT-PCR, qRT-PCR	RT-PCR for EBOV	I^2^ = 36%
Boltena et al., 2021 [22]	Soil-transmitted helminthiasis (Hookworm, *Ascaris lumbricoides*, *Trichuris trichiura*)	10	6633	Outpatients with suspected malaria	Ethiopia (SNNP, Amhara, Afar, Oromia regions)	*P. falciparum*, *P. vivax*, and mixed	Mainly microscopy	Mainly Kato-Katz	I^2^ = 97.54%
Del-Tejo et al., 2021 [23]	AIDS	17 (12 cross-sectional or longitudinal studies and 5 case reports)	1048 patients hospitalized for vivax malaria, 21 coinfected with HIV	Adults and children hospitalized	Mainly Africa (35.3%) and Asia (29.4%)	*P. vivax*	Microscopy (thick smear)	Rapid tests and immunoassays for HIV	Not applicable (no meta-analysis)
Kotepui et al., 2021 [24]	Trypanosomiasis	9	1523 patients with HAT (692 coinfected)	Patients with human African trypanosomiasis (HAT)	Angola, Kenya, Uganda, Tanzania, Sudan, Democratic Republic of Congo, Central African Republic, Ivory Coast, Equatorial Guinea, Republic of Congo	*P. falciparum* (predominant)	Microscopy or agglutination test	Microscopy (direct in blood, lymph node aspirate, or CSF) or molecular methods	I^2^ = 98.06%
Kotepui et al., 2020 [16]	Hepatitis B	22	22 studies (sample sizes varied by analysis: 7 studies for age analysis, 5 for gender analysis, 2 for liver function tests)	Febrile patients, blood donors, pregnant women, and outpatients	Nigeria, Brazil, Ghana, Central African Republic, Italy, Gambia	*P. falciparum*, *P. vivax*, mixed infections	Microscopy, RDT, PCR, ELISA	RDT, PCR, ELISA, or combination	I^2^ = 95.76%
Obebe et al., 2020 [15]	AIDS	58	23,911 (5001 cases of coinfection)	Adults and children	Sub-Saharan Africa	*P. falciparum*, *P. vivax*, *P. ovale*	Microscopy, RDT, PCR	Serological tests for HIV	I^2^ = 98.4%
Salam et al., 2018 [14]	Dengue, chikungunya	104	NR	Patients with fever or coinfection	South Asia, Africa, Southeast Asia, South America, North America, Caribbean, Middle East	*P. falciparum*, *P. vivax*, *P. ovale*, *P. knowlesi*	Microscopy, RDT, PCR	NS1, IgM/IgG ELISA, PCR	Not applicable (no meta-analysis)
Degarege et al., 2016 [5]	Soil-transmitted helminthiasis (STH)	11	7458	Children	Sub-Saharan Africa	*P. falciparum*	Microscopy	Stool examination (Kato Katz)	I^2^ = 36.8%
Church et al., 2014 [13]	Invasive bacteremia	25 (20 cohort analyses, 2 randomized controlled trials, and 3 epidemiological studies)	7208 children with severe malaria, 461 coinfected	Children	11 African countries	*P. falciparum*	Mainly microscopy	Blood culture	Not applicable (no meta-analysis)

Abbreviations: NR, not reported; I^2^, Higgins heterogeneity statistic; RDT, rapid diagnostic test; PCR, polymerase chain reaction; RT-PCR, reverse transcription polymerase chain reaction; qRT-PCR, quantitative reverse transcription polymerase chain reaction; ELISA, enzyme-linked immunosorbent assay; IgM, immunoglobulin M; IgG, immunoglobulin G; NS1, dengue non-structural protein 1; MAT, microscopic agglutination test; IFA, indirect fluorescent antibody assay; HIV, human immunodeficiency virus; AIDS, acquired immunodeficiency syndrome; COVID-19, coronavirus disease 2019; WHO, World Health Organization; IMCI, Integrated Management of Childhood Illness; EVD, Ebola virus disease; EBOV, Ebola virus; HAT, human African trypanosomiasis; CSF, cerebrospinal fluid; STH, soil-transmitted helminthiasis; DRC, Democratic Republic of the Congo; SNNP, Southern Nations, Nationalities, and Peoples Region (Ethiopia); *P.*, *Plasmodium*; *S.*, *Schistosoma*. Notes: (A), (B), (C), (D), and (E) following Wilairatana et al. denote separate publications by the same first author within the same year, ordered alphabetically. Asterisk (*) after Mohamed et al., 2024 indicates a narrative synthesis without pooled meta-analytical estimates.

**Table 2:** ROBIS assessment of the 21 systematic reviews included in the umbrella review.

Systematic Review (First Author and Year)	Study Eligibility Criteria	Identification and Selection of Studies	Data Collection and Appraisal	Synthesis and Findings	Overall Risk of Bias
Church, 2014 [13]	Low	Low	Low	High	High
Degarege, 2016 [5]	Low	Low	Low	Low	Low
Salam, 2018 [14]	Low	Low	Low	Low	Low
Obebe, 2020 [15]	Low	Low	Low	Low	Low
Kotepui, 2020 [16]	Low	Low	Low	Low	Low
Wilairatana (A), 2021 [17]	Low	Low	Low	Low	Low
Wilairatana (B), 2021 [18]	Low	Low	Low	Low	Low
Afolabi, 2021 [19]	Low	Low	Low	Low	Low
Wilairatana (C), 2021 [20]	Low	Low	Low	Low	Low
Edwards, 2021 [21]	Low	Low	Low	Low	Low
Boltena, 2021 [22]	Low	Low	Low	Low	Low
Del-Tejo, 2021 [23]	Low	Low	Low	Low	Low
Kotepui, 2021 [24]	Low	Low	Low	High	High
Mala, 2022 [6]	Low	Low	Low	High	High
Wilairatana (D), 2022 [25]	Low	Low	Low	High	High
Wilairatana (E), 2022 [26]	Low	Low	Low	High	High
Gebremariam 2023 [3]	Low	Low	Low	High	High
Setegn, 2024 [27]	Low	Low	Low	High	High
Cerilo-Filho, 2024 [28]	Low	Low	Low	Low	Low
Mohamed 2024 * [4]	Low	Low	Low	Low	Low
Abebe, 2025 [29]	Low	Low	Low	Low	Low

Notes: (A), (B), (C), (D), and (E) following Wilairatana denote separate publications by the same first author within the same year, ordered alphabetically. Asterisk (*) after Mohamed 2024 indicates a narrative synthesis without pooled meta-analytical estimates.

**Table 3:** Certainty of evidence for review-level malaria coinfection prevalence estimates.

Coinfection/Source Review	Prevalence or Range	Main Downgrading Domains	Overall Certainty
Schistosoma mansoni/Abebe 2025	17.39% (5.94–28.84)	HET, PREC, GEO, PB, OVL	Low
Schistosoma mansoni/Setegn 2024	10.496% (6.134–14.859)	RoB, HET, DIR, GEO	Very low
Dengue/arboviruses/Cerilo-Filho 2024	NR pooled; 746 coinfections	No pooled estimate, HET, OVL	Low
COVID-19/Mohamed 2024	Individual reports: 3.0%, 9.4%, 40.4%	No pooled estimate, PREC, GEO, OVL	Very low
Dengue/Gebremariam 2023	4.2% (3.0–6.0)	RoB, HET, GEO, OVL	Low
Severe pneumonia/Mala 2022	19% (12–26)	RoB, HET, DIR, GEO	Low
Bacteremia/Wilairatana 2022	7.6% (6.7–8.7)	RoB, HET, denominators	Low
Influenza/Wilairatana 2022	3% (2–5)	RoB, HET, DIR, GEO	Low
Helminths/Afolabi 2021	17.7% (12.7–23.2)	HET, DIR, GEO, OVL	Low
Leptospirosis/Wilairatana 2021	13% (9–18)	HET, DIR	Low
Typhoidal/non-typhoidal Salmonella/Wilairatana 2021	14% (9–19)	HET, DIR, GEO	Low
COVID-19/Wilairatana 2021	11% (4–18)	HET, PREC, OVL	Low
Ebola virus disease/Edwards 2021	21.7% (18.7–25.1)	GEO, malaria diagnostics varied	Moderate
Geohelminths/Boltena 2021	13% (9–17)	HET, GEO	Low
HIV/*P. vivax*/Del-Tejo 2021	2.0% (CI NR)	No meta-analysis, PREC, sparse evidence	Low
Human African trypanosomiasis/Kotepui 2021	50% (28–72)	RoB, HET, PREC, HAT-specific denominator	Very low
Hepatitis B/Kotepui 2020	6% (4–7)	HET, DIR, context variability	Low
HIV/AIDS/Obebe 2020	22.7% (18.0–28.1)	HET, GEO, DIR	Low
Dengue/chikungunya/Salam 2018	Regional ranges: 0.07–50%	No pooled estimate, HET, OVL	Low
Soil-transmitted helminths/Degarege 2016	Review-level outcomes; pooled estimate not consistently extractable	Older review, DIR, population limits	Low
Invasive bacteremia/Church 2014	6.4% (5.81–6.98) in severe malaria	RoB, hospitalized children, severe-malaria denominator	Low

Table notes: Domains used for downgrading: RoB, ROBIS risk of bias; HET, heterogeneity; DIR, diagnostic directness; PREC, precision; GEO, geographic representativeness; PB, publication-bias or small-study concern; OVL, primary-study overlap. Certainty ratings are conservative and based on review-level evidence.

## Data Availability

All data generated or analyzed during this umbrella review are included in this published article and its Supplementary Materials. The dataset consists of data extracted from the original systematic reviews, which are publicly available and cited in the manuscript. No additional unpublished data were created.

## Supplementary Materials

The following supporting information can be downloaded at https://www.mdpi.com/article/10.3390/tropicalmed11070206/s1. Supplementary Material S1: Search strategy by database, including the targeted corrective LILACS/BVS rerun; Supplementary Material S2: Completed PRISMA 2020 checklist; Supplementary Material S3: Primary-study overlap and corrected-covered-area assessment.
