# Supplementary material for: Malaria Coinfections Worldwide: An Umbrella Systematic Review of Prevalence and Epidemiological Patterns
Source: Trop Med Infect Dis. 2026 Jul 22;11(7):206. doi: 10.3390/tropicalmed11070206 (PMC13417142; doi:10.3390/tropicalmed11070206)
Supplement: Supplementary file 1 [file tropicalmed-11-00206-s001.zip › Supplementary Material S1. Search Strategy REVISED.pdf]

Supplementary Material S1. Search strategy by database.

PubMed

Table S1.1. Search strategy in PubMed.

Strategy	Search terms
#1	"Malaria"[Mesh] OR malaria[tiab] OR plasmodium[tiab]
#2	"Coinfection"[Mesh] OR coinfection*[tiab] OR "co-infection*"[tiab] OR "mixed infection"[tiab]
#3	"systematic review"[tiab] OR "systematic reviews"[tiab] OR "meta-analysis"[tiab] OR "meta-analyses"[tiab] OR "revisión sistemática"[tiab] OR "revisiones sistemáticas"[tiab] OR "metaanálisis"[tiab] OR "meta análisis"[tiab] OR "overview of reviews"[tiab] OR "umbrella review"[tiab] OR "umbrella reviews"[tiab]
#4	#1 AND #2 AND #3

Scopus

Table S1.2. Search strategy in Scopus.

Strategy	Search terms
#1	TITLE-ABS-KEY (malaria OR plasmodium)
#2	TITLE-ABS-KEY (coinfection* OR "co-infection*" OR "mixed infection")
#3	TITLE-ABS-KEY ("systematic review" OR "systematic reviews" OR meta-analysis OR "meta analysis" OR "umbrella review")
#4	#1 AND #2 AND #3

Web of Science

Table S1.3. Search strategy in Web of Science.

Strategy	Search terms
#1	TS=(malaria OR plasmodium)
#2	TS=(coinfection* OR "co-infection*" OR "mixed infection")
#3	TS=("systematic review" OR "systematic reviews" OR meta-analysis OR meta-analyses OR "meta analysis" OR "umbrella review" OR "overview of reviews")
#4	#1 AND #2 AND #3

EMBASE

Table S1.4. Search strategy in EMBASE.

Strategy	Search terms
#1	'malaria'/exp OR 'plasmodia infection' OR 'plasmodium infection' OR 'gametocytaemia' OR 'gametocytemia' OR 'infection by plasmodium' OR 'malaria' OR 'malaria infection' OR 'malaria transmission' OR 'malarial fever' OR 'malarial infection' OR 'marsh fever' OR 'paludism' OR 'plasmodial infection' OR 'plasmodiosis' OR 'swamp fever (malaria)'

#2	'coinfection'/exp OR 'co-infection' OR 'co-infections' OR 'co-occurring infection' OR 'coinfection' OR 'coinfections' OR 'concomitant infection' OR 'concomitant infections' OR 'concurrent infection' OR 'concurrent infections' OR 'concurring infection' OR 'mix infection' OR 'mixed infection' OR 'mixed infections' OR 'multi-infection' OR 'multi-microbial infection' OR 'multi-viral infection' OR 'multi-infection' OR 'multimicrobial infection' OR 'multiple infection' OR 'multiple infections' OR 'multiviral infection' OR 'poly-infection' OR 'poly-microbial infection' OR 'poly-viral infection' OR 'polyinfection' OR 'polymicrobial infection' OR 'polyviral infection' OR 'simultaneous infection' OR 'simultaneously occurring infection'
#3	'systematic review'/exp OR 'review, systematic' OR 'systematic review' OR 'meta analysis'/exp OR 'analysis, meta' OR 'meta analysis' OR 'meta-analysis' OR 'metaanalysis'
#4	#1 AND #2 AND #3

LILACS/BVS

Table S1.5. Search strategy in LILACS/BVS.

Targeted corrective LILACS/BVS rerun

After peer review, the LILACS/BVS component was rerun on 1 June 2026 in the BVS Regional Portal using harmonized systematic-review and meta-analysis terms. The rerun was restricted to the LILACS Plus collection and retrieved six records, corresponding to five unique records after one duplicate. Screening did not identify any additional eligible systematic review beyond those already included in the umbrella review.

Screening of unique records retrieved in the corrective LILACS/BVS rerun

Record	Decision	Reason
Worldwide distribution, symptoms and diagnosis of the coinfections between malaria and arboviral diseases: a systematic review	Included already	Appeared as a duplicate; corresponds to Cerilo-Filho 2024 already included.
Should we care about Plasmodium vivax and HIV coinfection? A systematic review and a cases series from the Brazilian Amazon	Included already	Corresponds to Del-Tejo 2021 already included.
Distribution, frequency and clinical presentation of leptospirosis and coinfections: a systematic review protocol	Excluded	Protocol; not a completed systematic review with malaria coinfection prevalence.
Effectiveness of spatially targeted interventions for control of HIV, tuberculosis, leprosy and malaria: a systematic review	Excluded	Intervention/geospatial review; not malaria coinfection prevalence.
Epidemiology of acute febrile illness in Latin America	Excluded	Broad febrile-illness review; not a malaria coinfection systematic review with extractable prevalence.

Item	Result
Platform	BVS Regional Portal

Collection searched	LILACS Plus
Date of corrective rerun	1 June 2026
Records retrieved	6
Unique records after duplicate removal	5
Additional eligible systematic reviews	0
Effect on final included reviews	None
Effect on original PRISMA flow	None; reported as targeted corrective rerun

**Strategy**	**Search terms**
#1	tw:(malaria OR plasmodium)
#2	tw:(coinfection* OR "co-infection*" OR "mixed infection" OR "infeccion mixta" OR "infecciones combinadas" OR coinfeccao OR "infeccao mista")
#3	tw:("systematic review" OR "systematic reviews" OR "revision sistematica" OR "revisao sistematica" OR "meta-analysis" OR "meta analysis" OR metaanalisis OR "meta analisis" OR metanalise OR "meta-analise" OR "umbrella review" OR "overview of reviews" OR "overview of systematic reviews")
#4	#1 AND #2 AND #3
