# Supplementary material for: Malaria Coinfections Worldwide: An Umbrella Systematic Review of Prevalence and Epidemiological Patterns
Source: Trop Med Infect Dis. 2026 Jul 22;11(7):206. doi: 10.3390/tropicalmed11070206 (PMC13417142; doi:10.3390/tropicalmed11070206)
Supplement: Supplementary file 1 [file tropicalmed-11-00206-s001.zip › Supplementary Material S2. PRISMA 2020 Checklist REVISED.pdf]

Supplementary Material S2. Completed PRISMA 2020 Checklist

Note: The checklist was completed for an umbrella systematic review of systematic reviews. Items were mapped to the manuscript sections where the corresponding information is reported.

PRISMA 2020 item	Topic	Reporting item (paraphrased)	Location in manuscript / comments
1	Title	Identify the report as a systematic review.	Title changed to include “Umbrella Systematic Review”; article type changed to “Systematic Review”.
2	Abstract	Provide a structured summary covering rationale, objectives, eligibility criteria, sources, methods, results, limitations, conclusions, and registration information when applicable.	Abstract. Registration status is stated in Methods; limitation added in Section 4.7.
3	Rationale	Describe the rationale for the review in the context of existing evidence.	Introduction, paragraphs 1–3.
4	Objectives	State the review objectives or questions clearly.	Introduction, final paragraph.
5	Eligibility criteria	Specify inclusion and exclusion criteria and how studies were grouped for synthesis.	Sections 2.3 and 2.4.
6	Information sources	Specify databases, registers, websites, organizations, reference lists, and dates of search or consultation.	Section 2.2; Supplementary Material 1. The original search covered records from 1 January 2000 to March 2026. A targeted corrective LILACS/BVS rerun was performed on 1 June 2026 and is

			reported in Supplementary Material 1.
7	Search strategy	Present full search strategies for all databases, registers, and websites.	Section 2.2; Supplementary Material 1. Full original database strategies and the corrective LILACS/BVS rerun strategy are reported.
8	Selection process	Describe how records and reports were screened and selected, including number of reviewers and conflict resolution.	Section 2.5.
9	Data collection process	Describe how data were extracted and verified, including number of reviewers and conflict resolution.	Section 2.6.
10a	Data items	List and define all outcomes for which data were sought.	Sections 2.3 and 2.6.
10b	Other variables	List and define additional variables for which data were sought.	Section 2.6.
11	Risk of bias assessment	Describe the tool and process used to assess risk of bias of included studies.	Section 2.7.
12	Effect measures	Specify measures used for each outcome.	Sections 2.6 and 2.8; prevalence and 95% confidence intervals were extracted.
13a	Synthesis methods: eligibility for synthesis	Describe how studies were judged eligible for each synthesis.	Sections 2.3, 2.4, and 2.8.
13b	Synthesis methods: data preparation	Describe data preparation or transformation methods.	Sections 2.6 and 2.8.
13c	Synthesis methods:	Describe methods	Section 2.8; Tables 1–

	tabulation/visual display	used to tabulate or visually display results.	2; Figures 1–2.
13d	Synthesis methods: synthesis approach	Describe synthesis methods used.	Section 2.8. Narrative and descriptive review-level synthesis; no de novo pooled meta-analysis was conducted. Primary-study overlap and certainty of evidence were assessed using CCA and an adapted GRADE framework.
13e	Synthesis methods: heterogeneity exploration	Describe methods used to explore possible causes of heterogeneity.	Sections 2.8, 3.3, and 3.5. Heterogeneity was interpreted at review level and contextualized by diagnostic methods, population, geography, and pathogen group.
13f	Synthesis methods: sensitivity analyses	Describe sensitivity analyses when performed.	Not applicable; this umbrella review extracted estimates from published systematic reviews rather than conducting a de novo meta-analysis.
14	Reporting bias assessment	Describe methods for assessing risk of bias due to missing results.	ROBIS was used to assess risk of bias in included reviews. Publication-bias or small-study concerns were also considered in the adapted certainty-of-evidence assessment and limitations.
15	Certainty assessment	Describe methods for assessing certainty or confidence in the body of evidence.	Certainty of evidence for each review-level prevalence estimate

			was assessed using an adapted GRADE framework for prevalence evidence, considering ROBIS risk of bias, heterogeneity, diagnostic directness, precision, geographic representativeness, and publication-bias or small-study concerns. Results are reported in Table 3.
16a	Study selection results	Report the number of records/reports at each stage and reasons for exclusions.	Section 3.1; Figure 1.
16b	Studies that appear to meet criteria but were excluded	Cite studies that appeared eligible but were excluded and explain why.	Section 3.1 summarizes exclusion categories; individual excluded full texts are not listed separately.
17	Study characteristics	Present characteristics of each included study.	Section 3.2; Table 1.
18	Risk of bias in studies	Present risk-of-bias assessments for included studies.	Section 3.4; Table 2.
19	Results of individual studies	Present results for each included study or synthesis.	Section 3.5; Figure 2; Table 1.
20a	Results of syntheses	Summarize results for each synthesis.	Section 3.5; Figure 2.
20b	Heterogeneity results	Present results of heterogeneity analyses.	Section 3.3; Table 1.
20c	Sensitivity analyses	Present results of sensitivity analyses when performed.	Not applicable to de novo meta-analysis. A sensitivity-style broad CCA matrix was reported for

			malaria-helminth/STH reviews where objectives were related but not identical.
20d	Subgroup/additional analyses	Present results of subgroup or additional analyses when performed.	Sections 3.5 and 3.6; Figure 3. Additional analyses included CCA by pathogen cluster, certainty of evidence, predominant infection-pattern categories, and review-level geographic coverage.
21	Reporting biases	Present assessments of risk of bias due to missing results.	Addressed through ROBIS synthesis/findings domain, adapted certainty assessment, and limitations; Sections 3.4, 3.5, and 4.7.
22	Certainty of evidence	Present certainty or confidence assessments for each outcome when performed.	Certainty of evidence was assessed using the adapted GRADE framework and is reported in Table 3.
23a	Discussion: general interpretation	Interpret results in context of other evidence.	Discussion Sections 4.1–4.6.
23b	Discussion: limitations of evidence	Discuss limitations of included evidence.	Sections 4.1 and 4.7.
23c	Discussion: limitations of review process	Discuss limitations of the review methods.	Section 4.7.
23d	Discussion: implications	Discuss implications for practice, policy, and future research.	Sections 4.5, 4.6, and Conclusions.
24a	Registration and protocol	Provide registration information or state that the review was not registered.	Section 2.1 states that the review was not prospectively registered in PROSPERO or

			another registry; this is addressed as a transparency limitation in Section 4.7.
24b	Protocol availability	Indicate where the protocol can be accessed or state that none is available.	No registered public protocol is available; stated in Section 2.1 and addressed in Section 4.7.
24c	Amendments	Describe amendments to protocol or state not applicable.	Not applicable because no prospective registered protocol was available.
25	Support	Describe financial or non-financial support.	Funding statement and Acknowledgments.
26	Competing interests	Declare competing interests.	Conflicts of Interest statement.
27	Availability of data/materials/code	Report availability of data, materials, and code.	Data Availability Statement; Supplementary Materials 1-3. CCA calculations and search rerun decisions are available in the supplementary materials.

Abbreviations: PRISMA, Preferred Reporting Items for Systematic Reviews and Meta-Analyses; ROBIS, Risk of Bias in Systematic Reviews; SR, systematic review.
