# Supplementary material for: Malaria Coinfections Worldwide: An Umbrella Systematic Review of Prevalence and Epidemiological Patterns
Source: Trop Med Infect Dis. 2026 Jul 22;11(7):206. doi: 10.3390/tropicalmed11070206 (PMC13417142; doi:10.3390/tropicalmed11070206)
Supplement: Supplementary file 1 [file tropicalmed-11-00206-s001.zip › Supplementary Material S3. CCA Overlap REVISED.pdf]

Supplementary Material S3. Primary-study overlap and corrected covered area assessment

This supplement reports corrected covered area (CCA) calculations for clusters of included systematic reviews that shared comparable pathogen-specific objectives and for which primary-study lists could be identified. CCA was not calculated for singleton clusters, non-comparable review questions, or review groups with incomplete primary-study lists.

Formula: CCA = (N - r)/[(r x c) - r], where N is the total number of primary-study occurrences across reviews, r is the number of unique primary studies, and c is the number of systematic reviews in the matrix. Interpretation: 0-5% = slight, >5-10% = moderate, >10-15% = high, and >15% = very high overlap.

Comparable cluster	Reviews included	c	N	r	CCA	Interpretation
Malaria-dengue (DENV subset)	Gebremariam 2023; Cerilo-Filho 2024 DENV rows; Salam 2018 DENV rows	3	128	80	30.0%	Very high
Malaria-Schistosoma mansoni	Abebe 2025; Setegn 2024	2	24	21	14.3%	High
Malaria-COVID-19	Wilairatana 2021; Mohamed 2024	2	31	30	3.3%	Slight
Malaria-helminths/STH sensitivity matrix	Afolabi 2021; Degarege 2016; Boltena 2021	3	76	64	9.4%	Moderate

Repeated primary studies identified

Matrix	Comparison	Repeated primary studies
Malaria-dengue	Triple overlap	Ayorinde 2016; Chipwaza 2014; Kolawole 2017; Oyero 2014; Sow 2016.
Malaria-dengue	Cerilo-Filho 2024 + Salam 2018 additional	Abbasi 2009; Ahmad 2016; Alam 2013; Arya 2005; Assir 2012; Ayuthaya 2014; Bhagat 2014; Carme 2009; Chander 2009; Charrel 2005; Che Rahim 2017; Chong 2017; Deresinski 2006; Epelboin 2012; Faruque 2012; Kaushik 2007; Lupi 2016; Magalhaes 2012; Magalhaes 2014; McGready 2010; Mittal 2015; Mueller 2014; Mushtaq 2013; Pande 2013; Raja 2016; Rao 2016; Saksena 2017; Santana 2010; Serre 2015; Shah 2017; Singh 2014; Stoler 2015; Thangaratham 2006; Ward 2006; Yong 2012.
Malaria-dengue	Cerilo-Filho 2024 + Gebremariam 2023 additional	Nkenfou 2021; Onyedibe 2018.
Malaria-dengue	Salam 2018 + Gebremariam 2023 additional	Baba 2013.
Malaria-Schistosoma mansoni	Abebe 2025 + Setegn 2024	Dufera 2016; Getie 2015; Hailu 2018.
Malaria-COVID-19	Wilairatana 2021 + Mohamed 2024	Mahajan health-care worker cohort in India; appears as Mahajan 2021 in Wilairatana and as Mahajan 2020 in Mohamed.
Malaria-helminths/STH	Afolabi 2021 + Degarege 2016	Abanyie 2013; Achidi 2008; Kinunghi 2014; Kirwan 2010; Mazigo 2010; Mboera 2011; Nkuo-Akenji 2006; Righetti 2012; Roussilhon 2010.
Malaria-helminths/STH	Afolabi 2021 + Boltena 2021	Alemu 2012; Degarege 2014; Deribew 2013.

Malaria-helminths/STH	Degarege 2016 + Boltena 2021	No shared primary studies identified.

Clusters not calculated

CCA was not calculated for bacteremia/Salmonella, HIV/AIDS, respiratory infections, leptospirosis, hepatitis B, trypanosomiasis, Ebola virus disease, or other singleton/non-comparable clusters because the required criteria for a meaningful CCA matrix were not met.

Methodological source: Pieper D, Antoine SL, Mathes T, Neugebauer EAM, Eikermann M. Systematic review finds overlapping reviews were not mentioned in every other overview. Journal of Clinical Epidemiology. 2014;67(4):368-375. doi:10.1016/j.jclinepi.2013.11.007.
